# Whole Brain Mapping of Long-Range Direct Input to Glutamatergic and GABAergic Neurons in Motor Cortex

**DOI:** 10.3389/fnana.2019.00044

**Published:** 2019-04-17

**Authors:** Pan Luo, Anan Li, Yanxiao Zheng, Yutong Han, Jiaojiao Tian, Zhengchao Xu, Hui Gong, Xiangning Li

**Affiliations:** ^1^Britton Chance Center for Biomedical Photonics, Wuhan National Laboratory for Optoelectronics-Huazhong University of Science and Technology, Wuhan, China; ^2^MoE Key Laboratory for Biomedical Photonics, School of Engineering Sciences, Huazhong University of Science and Technology, Wuhan, China; ^3^HUST-Suzhou Institute for Brainsmatics, Suzhou, China

**Keywords:** motor cortex, whole brain, long-range input, distinct distribution, 3D

## Abstract

Long-range neuronal circuits play an important role in motor and sensory information processing. Determining direct synaptic inputs of excited and inhibited neurons is important for understanding the circuit mechanisms involved in regulating movement. Here, we used the monosynaptic rabies tracing technique, combined with fluorescent micro-optical sectional tomography, to characterize the brain-wide input to the motor cortex (MC). The whole brain dataset showed that the main excited and inhibited neurons in the MC received inputs from similar brain regions with a quantitative difference. With 3D reconstruction we found that the distribution of input neurons, that target the primary and secondary MC, had different patterns. In the cortex, the neurons projecting to the primary MC mainly distributed in the lateral and anterior portion, while those to the secondary MC distributed in the medial and posterior portion. The input neurons in the subcortical areas also showed the topographic shift model, as in the thalamus, the neurons distributed as outer and inner shells while the neurons in the claustrum and amygdala were in the ventral and dorsal part, respectively. These results lay the anatomical foundation to understanding the organized pattern of motor circuits and the functional differences between the primary and secondary MC.

## Introduction

The motor cortex (MC) plays a crucial role in the generation and control of movement and motor learning (Sanes and Donoghue, 2000; Tanji, 2001; Peters et al., 2017). Dysfunction of the MC can cause many neurological diseases, such as Parkinson’s disease, amyotrophic lateral sclerosis, Huntington’s disease and Alzheimer’s disease (Ferreri et al., 2011; Shepherd, 2013). There are two functional subregions in the MC, primary motor cortex (MOp) and the secondary motor cortex (MOs) (Sul et al., 2011; Guo J. Z. et al., 2015). A study based on intracortical microstimulation revealed that stimulation of the MOp and MOs induced movements of the forelimbs and whiskers in mice, respectively (Tennant et al., 2011). The MOs is more involved in cognitive-related motor control processing, such as motor decision, motor planning, motor learning, and spatial memory (Barthas and Kwan, 2017). The functional differences between the MOp and MOs rely on integrating information from upstream areas and sending information to downstream areas through dendrites and axons. Investigation of the connectivity patterns, including the input and output circuits, are essential to dissect the diverse functions of these subregions. With viral neuronaltracing, the output circuit pattern MC has been well-identified, in which the projections from the MOp and MOs showed unique and separate tract pathways, despite targeting similar areas (Jeong et al., 2016). However, the organization patterns of the upstream circuits in the whole brain, especially the direct input to the subregions of the MC, are not clear at present.

Previous studies revealed that the MC integrates inputs from many brain areas, such as the orbital cortex, primary somatosensory cortex (especially the barrel field, SSp-bfd), secondary somatosensory cortex (SSs), ventrolateral thalamic nucleus (VL) and the ventromedial thalamic nucleus (VM) in the thalamus, and basal forebrain (Hooks et al., 2013; Zingg et al., 2014; Zaborszky et al., 2015). But these studies were based on tracing methods that cannot identify the whole brain input to the specific type of neurons, while the neurons in the MC can be divided into two main categories: glutamatergic neurons and GABAergic neurons (Huang, 2014). The former sends long-range axons to other areas for innervation, and the latter mainly establishes the connection in the local area to carry on the regulation, accounting for about 20% of the total cortical neurons. The monosynaptic rabies tracing technique has been widely used to characterize the presynaptic inputs of desired starter neurons with high accuracy and efficiency (Wickersham et al., 2007a; Wall et al., 2010; Ogawa et al., 2014). The inputs to glutamatergic neurons and three subtypes of GABAergic neurons in the MC has been revealed (Zhang et al., 2016). But structure information of the upstream circuits of the subregions of the MC, including the MOp and MOs, especially the whole-brain input to different types of neurons, remains indistinct.

To map the whole brain inputs to glutamatergic and GABAergic neurons in the MOp and MOs, here, we used a dual-color monosynaptic rabies tracing technique combined with fluorescent Micro-Optical Sectional Tomography (fMOST) (Gong et al., 2016) and performed systematic analyses and comparisons.

## Materials and Methods

### Animal

Adult (2–4 months) C57BL/6J mice Thy1-cre mice (JAX: 006143) and Vgat-cre mice (JAX: 028862) were used in this study, targeting glutamatergic neurons (Campsall et al., 2002; Yang H. et al., 2016; Yang Y. et al., 2016) and GABAergic neurons (Vong et al., 2011; Chen et al., 2018), respectively. All animals were housed in normal cages in an environment with a 12-h light/dark cycle and were free to get enough food and water. All animal experiments were approved by the Animal Ethics Committee of Huazhong University of Science and Technology.

### Surgery and Stereotaxic Injection of Virus

The mice were intraperitoneally injected with 1% pentobarbital sodium for anesthesia. The anesthetized mice were attached to a mouse adapter. A drill was used to make a small hole in the skull above the target area. A virus was injected into the target area using a pressure injection pump (Nanoject II: Drummond Scientific, Co., Broomall, PA, United States). The wound was cleaned alternately with iodine and 75% alcohol to prevent inflammation.

In this study, we used a monosynaptic rabies tracing technique to label the whole-brain inputs to specific cell types in the MOp and MOs simultaneously in a same transgenic mouse. First, 150 nl AAV helper mixtures were injected into the ipsilateral MOp (AP:1.54 mm, ML:1.70 mm, DV:-1.50 mm) and MOs (AP:1.54 mm, ML:0.50 mm, DV:-1.35 mm) in Thy1-cre or Vgat-cre mice respectively, mixed with rAAV2/9-Ef1α-DIO-BFP-2a-TVA-WPRE-pA and rAAV2/9-Ef1α-DIO-RG-WPRE-pA as the ratio of 1:2. Three weeks later, 300 nl RV-ΔG-EnVA-EGFP and RV-ΔG-EnVA-Dsred were injected into the two subregions of the MC respectively. One week later, the mice were perfused. The titer of both AAVs is 2.00E+12 vg/ml, while the titer of RV is 2.00E+8 IFU/ml. The virus used was produced by BrainVTA. The amplification origins of RVs were from SADΔG-EGFP (EnvA) (Wickersham et al., 2007b; Osakada et al., 2011). The AAV virus vectors were constructed by BrainVTA. The coding region of the TVA element and RG element were obtained from the AAV-EF1a-FLEX-GT plasmid (Addgene plasmid 26198) and pAAV-EF1a-FLEX-RG plasmid (Addgene plasmid 98221) respectively, and were separately constructed into the DIO cassette of the plasmid pAAV-EF1a-DIO-hChR2 (H134R)-EYFP (Addgene plasmid 20298) (Hu et al., 2016).

During the stereotaxic injection, we set the Bregma as the zero point of the stereotactic coordinate. Briefly, Bregma is the junction of the coronal suture and the sagittal suture of the skull, and Lambda is the point of intersection of the bestfit lines passing through the sagittal suture and the left and right portions of the lambdoid suture. When Bregma and Lambda are on the same level and the left and right hemispheres are symmetric in the plane with the center line, the Bregma was set as the zero point.

### Histology

All histological procedures followed previous reports (Gong et al., 2016; Ren et al., 2018). The anesthetized mice received cardiac perfusion using 0.01 M phosphate-buffered saline (PBS, Sigma-Aldrich, United States) and 4% paraformaldehyde (PFA, Sigma-Aldrich, United States) in 0.01 M PBS. Brains were separated and then post-fixed with 4% PFA for 48 h. After fixation, the brain tissues were rinsed with PBS overnight.

To perform three-dimensional reconstruction analysis, some brains were embedded with GMA resin for whole brain imaging with the fMOST system. Briefly, the brains were dehydrated in a graded ethanol series (50, 70, and 95% ethanol, changing from one concentration to the next every 1 h at 4°C). Then, the brains were immersed in a graded glycol methacrylate (GMA, Ted Pella, Inc., Redding, CA, United States): 70, 85, and 100% GMA for 2 h each, and 100% GMA overnight at 4°C, and into a pre-polymerization of GMA for 3 days for penetration. Last, the samples were polymerized in a vacuum oven at 48°C for 24 h.

### Imaging

To detect labeling signals, some brains were manually sectioned with 50 μm coronal slices using the vibrating slicer (Leica 1200S). Then, the slices were imaged using confocal microscopy (Zeiss LSM710).

For whole brain imaging, the brains were embedded with GMA resin and sectioned and imaged automatically and continuously using the fMOST system, with the voxel resolution of 0.32 μm × 0.32 μm × 2 μm (Gong et al., 2016). We processed the images following the procedures used in our previous work (Gong et al., 2016; Li et al., 2017, 2018). For the whole brain dataset, we uploaded the images into Amira software (v5.2.2, Mercury Computer Systems, San Diego, CA, United States) and Fiji (NIH) to perform the basic operations, including extraction of areas of interest, resampling, maximum projection etc. In order to distinguish the brain region boundary, the down sampling data were registered into the Allen Reference Atlas (the voxel resolution of 10 μm × 10 μm × 10 μm) (Kuan et al., 2015). For registration, we performed the following steps: (1) image preprocessing, including uneven illumination correction, image contour outside background noise removal; (2) extracting regional features of anatomically invariants in the whole brain such as the ventricles, the hippocampus, the corpus callosum, etc.; (3) used the current advanced gray-level based registration algorithm (SyN) to register the extracted features, and to obtain the corresponding relationship between the image dataset and Allen CCFv3 brain atlas. (4) the registration parameters were applied to the whole dataset. For the three-dimensional (3D) reconstruction analysis and presentation, we extracted the cell body information by NeuroGPS software (Quan et al., 2016) and placed them in the corresponding 3D brain region contour.

### Statistical Analysis

We used 50 μm of maximum fluorescence signal projection or 50 μm sections from eight brains to perform cell counting using Fiji software. For cell counting in each area, we imported the image to the Fiji software and used its cell counter module to perform the manual cell count. Every two slices or sections were counted. We counted all the long-range upstream brain regions (upstream brain regions except for injection site -namely the MOp and MOs at the same hemisphere with the injection sites). If the number of cell bodies in any region of any sample was over 10, we regarded it as valid and there were 48 valid upstream regions. Then, we compared the percentage of input neurons of each valid region as the total input neurons with one-way ANOVA followed by Tukey’s Honest Significant Difference test using SPSS (version 13.0) (Beier et al., 2015). To quantify the similarity in input patterns, we calculated Pearson’s correlation coefficients.

## Results

### Monosynaptic Inputs to Glutamatergic and GABAergic Neurons in Subdomains of MC

**FIGURE 1:**
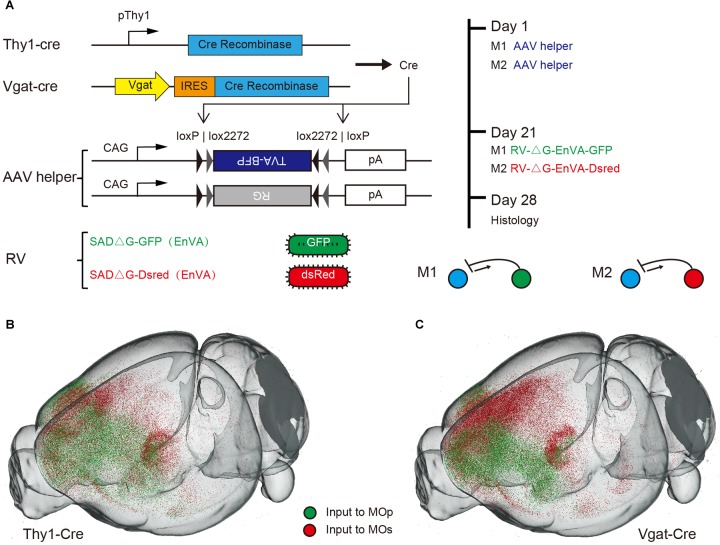
Monosynaptic rabies tracing the inputs to specific neurons in MC. **(A)** Schematic showing the dual-color monosynaptic rabies tracing technique with cre dependent mice (Thy1-cre mice and Vgat-cre mice) and viral vectors. **(B,C)** 3D reconstruction of the whole brain dataset acquired using the fMOST system. Green dots denote neurons projecting to the MOp while red dots denote neurons projecting to the MOs.

For mapping the whole brain monosynaptic input to the MC, we used viral neuronal tracing with an AAV helper and the modified rabies (RV), which could perform the direct monosynaptic inputs tracing. The RV pseudo typed with the avian sarcoma leucosis virus glycoprotein EnvA, can only infect cells expressing a cognate receptor (TVA protein) and requires the rabies virus to envelope the glycoprotein (RG) to spread retrogradely into the presynaptic cells. To compare the inputs to different categories of MC, we used the Thy1-cre mice and Vgat-cre mice respectively, that expressed a cre recombinant enzyme in glutamatergic or GABAergic neurons in all layers of cortex (Figure 1A). Combining cre-line mice with the cre-dependent AAV helper of the RV system, we could perform the inputs tracing, targeting the presynaptic neurons projecting to the specific type of neuron.

To investigate the input differences between the MOp and MOs, we selected two representative sites of these regions. One for forelimb motor controlling and the other for vibrissa motor controlling (Jeong et al., 2016). The dual-color monosynaptic rabies tracing technique was performed to label the input neurons of these sites respectively. In simple terms, an AAV helper that could express the TVA and G protein in specific neurons were injected in ipsilateral MOp and MOs of cre-line mice. Three weeks later, the RV expressing GFP (RV-ΔG-EnVA-GFP) or Dsred (RV-ΔG-EnVA-Dsred) were injected into the MOp and MOs respectively (Figure 1A). Thus, we could label monosynaptic inputs to specific type neurons of these regions in individual brains simultaneously. To confirm that the starter cells restricted to the injection site area, we injected one type of rabies virus in individual mice (Supplementary Figure S1) while the control was injected with TVA-BFP and RV, without RG in cre-line mice (Supplementary Figure S2). And the continuous coronals around the injection sites of dual-color RV also indicated that there was almost no crosstalk between the two RVs injected in the MOp and MOs (Supplementary Figure S3). With immunohistochemical staining, we further confirmed the cell specificity of starter cells in Thy1-cre mice (Supplementary Figure S4). We also performed control experiments in C57BL/6J mice to ensure that the leaked labeling had no effect on the analysis of our experiments (Supplementary Figure S5).

To analyze the whole brain inputs from the perspective of a 3D space, the brains were embedded with GMA resin, then sectioned and imaged automatically and continuously using the fMOST system. Through the 3D reconstruction, we could observe the whole brain input distribution characteristics of the MOp and MOs in a three-dimensional space (Figure 1B,C). Obviously, we found that the upstream neurons of both glutamatergic and GABAergic neurons, were mainly concentrated in the cortical area near the injection site and the thalamic area.

**FIGURE 2:**
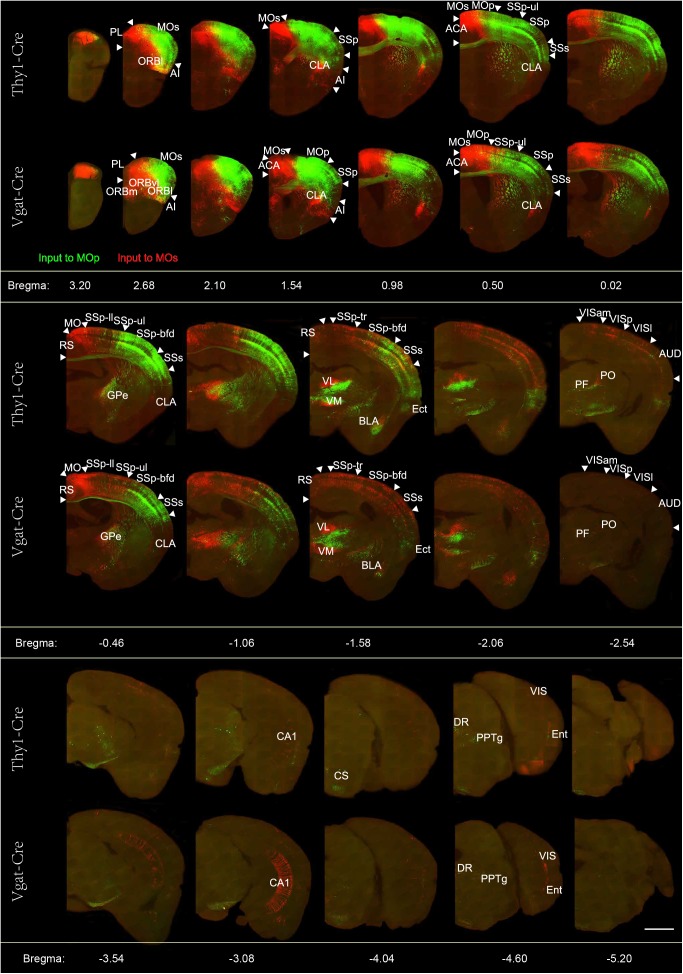
Representative images of selected regions with monosynaptic inputs to the glutamatergic and GABAergic neurons in the motor cortex. Continuous coronal view of maximum intensity projection of the Z stack (50 μm) across the entire brain. RV-labeled neurons identified by the green signal show the neurons projecting to the Mop, while the red signal indicates the neurons projecting to the MOs. Scale bar = 1 mm.

In order to show the signal distribution characteristics of these input brain regions more clearly, we performed the 50 μm maximum intensity projection on continuous 2 μm images (Figure 2). The GFP labeling neurons indicate populations projecting to the MOp, and the Dsred labeling neurons indicate populations projecting to the MOs. Overall, we found that the whole brain input distribution of glutamatergic neurons and GABAergic neurons are similar in the same subregions of the MC. But the whole brain input distribution to glutamatergic neurons or GABAergic neurons are basically separated in different subregions of the MC. In general, we observed that the glutamatergic neurons and GABAergic neurons in the MOp receive projections from the cortical plate (ORB, SSp-ul/m, SSp-bfd, SSs, ECT), cortical subplate (CLA, BLA), thalamus (VAL, VM, PO, PF), pallidum (NDB, SI, PALd), and the midbrain (VTA, DR, CS, PPN). The glutamatergic neurons and GABAergic neurons in the MOs receive projections from the cortical plate (ORB, ACA, RSP, SSp-ll /tr, SSp-bfd, SSs, ECT, hippocampus), cortical subplate (CLA, BLA), thalamus (AM, MD, LP, VAL, VM, PO, PF), pallidum (MS, NDB, SI, PALd), and the midbrain (VTA, DR, CS, PPN). The distribution of upstream neurons labeled with single RV is similar to the results of dual-color RVs (Supplementary Figure S6). The abbreviations of brain regions are summarized in Supplementary Table S1.

### Quantitative Comparison of Inputs to Specific Cell Types Between Subdomains of MC

**FIGURE 3:**
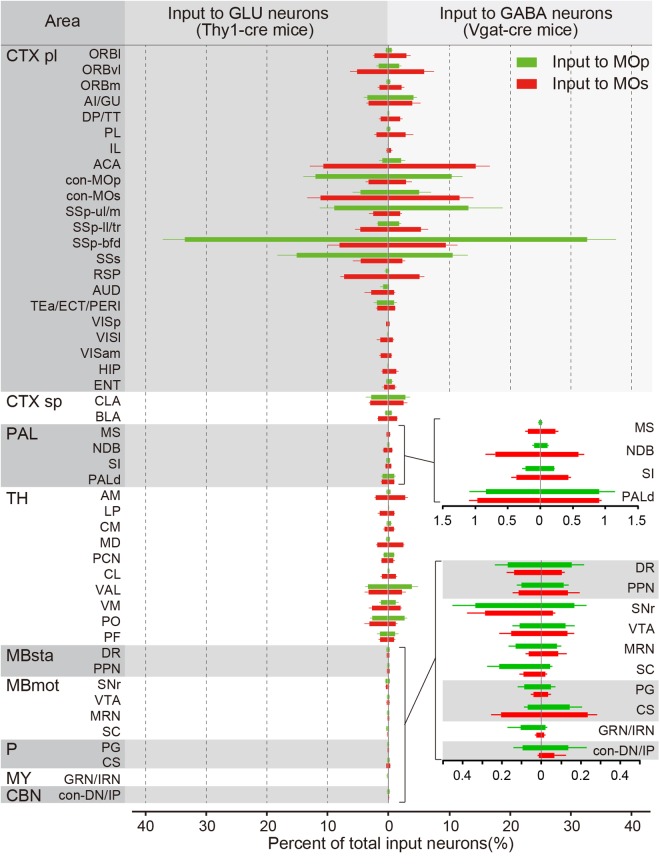
The proportions of the whole-brain input to the glutamatergic and GABAergic neurons in the MOp and MOs. The percentage of inputs from 48 upstream brain regions across the whole brain. (Left) Monosynaptic inputs to the glutamatergic neurons (GLU) in the MOp (green) and MOs (red). (Right) Monosynaptic inputs to GABAergic neurons (GABA) in the MOp (green) and MOs (red). Details of proportions of subregions in the pallidum (superior) and the midbrain, hindbrain, and the cerebellar nuclei (inferior) are shown in the lower right. The abbreviations of brain regions are provided in Supplementary Table S1. Mean ± SEM. Vgat-cre mice, *n* = 4; Thy1-cre mice, *n* = 4.

To quantitatively compare the input distribution of different subregions, we counted input neurons in each upstream region and normalized to the total number of the whole brain. To accurately distinguish the brain regions, we registered consecutive three-dimensional data to the Allen Reference Atlas. As shown in Figure 3, most areas projecting to the MOp also project to the MOs. However, there are a small number of brain regions such as the cortex (DP/TT, RSP, VISam, VISl), thalamus (LP, MD, CL) and the HIP that almost only project to the MOs, and not the MOp. In addition, the MOp receives convergent inputs. For example, there are only four of 48 areas with an input percentage greater than 5% for inputs to the glutamatergic neurons in the MOp, only nine of 48 areas with an input percentage greater than 2%, and the total percentage of these four main input areas is 69.2 ± 6.9%. Relatively, the MOs receives divergent inputs, with five of 48 areas with an input percentage greater than 5% for inputs to the glutamatergic neurons in the MOs, with 16 of 48 areas with an input percentage greater than 2%, and the total percentage of these five main input areas is 41.9 ± 7.4%. In detail, the MOp receives a large projection from both the SSp-bfd and SSs, from which the MOs receives a relatively small projection; the MOs receives a certain number of projections from the ACA and RSP, but the MOp receives almost no projection from these sites; Both the MOp and the MOs receives a small number of innervations from modulatory nuclei in the pallidus and midbrain.

### Distinct Input to the MOp and MOs but Similar to the Glutamatergic and GABAergic Neurons

**FIGURE 4:**
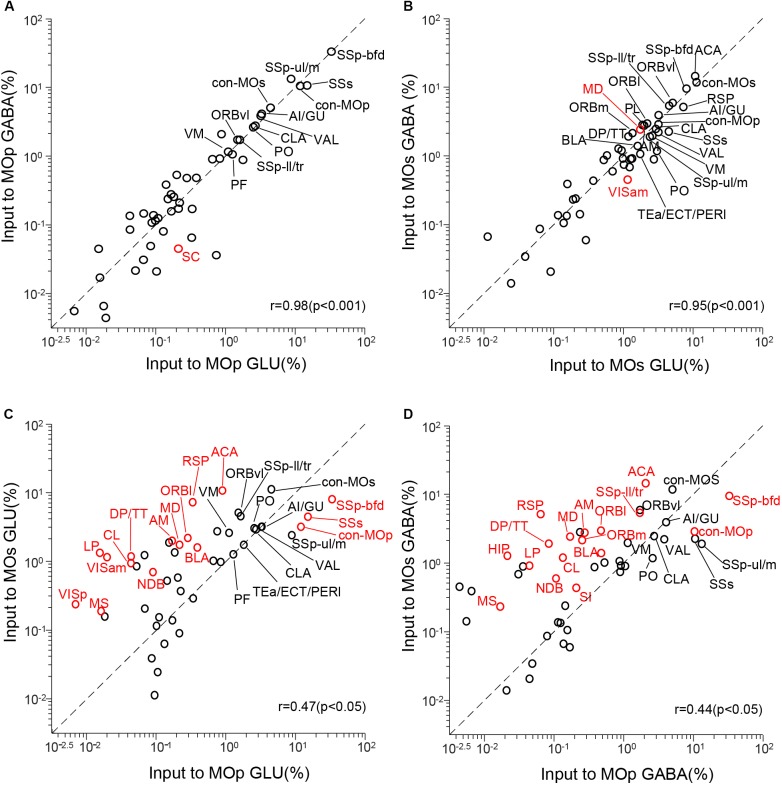
Comparisons of monosynaptic inputs to motor cortex. **(A)** Comparison between inputs to the glutamatergic neurons (GLU) and GABAergic neurons (GABA) in the MOp. **(B)** Comparison between inputs to the GLU and GABA in the MOs. **(C)** Comparison between inputs to the GLU in the MOp and MOs. **(D)** Comparison between inputs to the GABA in the MOp and MOs. Values are the means of the percentage of the total inputs from each region. Red circles indicate significant differences (*P* < 0.05, corrections for multiple comparisons with Tukey’s Honest Significant Difference test). r: Pearson’s correlation coefficient.

To quantify the correlation between these four groups of inputs to the MC, we conducted a correlation analysis (Figure 4). We compared the inputs of glutamatergic neurons and GABAergic neurons in the same brain region and compared the inputs of the same types of neurons in the MOp and MOs. Each circle in the scatter plot represents one brain region (significant differences in red, *P* < 0.05), and the diagonal line represents the same input proportion for each pair (Ogawa et al., 2014).

The correlation coefficient of input to the glutamatergic and GABAergic neurons in the MOp is 0.98 (*P* < 0.001), and only one of 48 upstream regions (SC, *P* = 0.017) showed a significant difference (Figure 4A). As for the input to the glutamatergic and GABAergic neurons in the MOs, the correlation coefficient is 0.95 (*P* < 0.001), and two upstream regions (MD, *P* = 0.038; VISm, *P* = 0.014) showed a significant difference (Figure 4B). These results show that the input patterns to the glutamatergic and GABAergic neurons in the MC are similar. When we compared the input to the glutamatergic neurons in the MOp and MOs, the correlation coefficient is 0.47 (*P* < 0.05), and 16 upstream regions show a significant difference (Figure 4C). The correlation coefficient of input to the GABAergic neurons in the MOp and MOs is 0.44 (*P* < 0.05), and 17 areas show a significant difference (Figure 4D). All these data indicate that the input patterns of the same type of neurons in different subregions of the MC are of great distinction. We then focused on the comparison of input patterns between different subregions.

### Region-Specific Projection to the MOp and MOs

**FIGURE 5:**
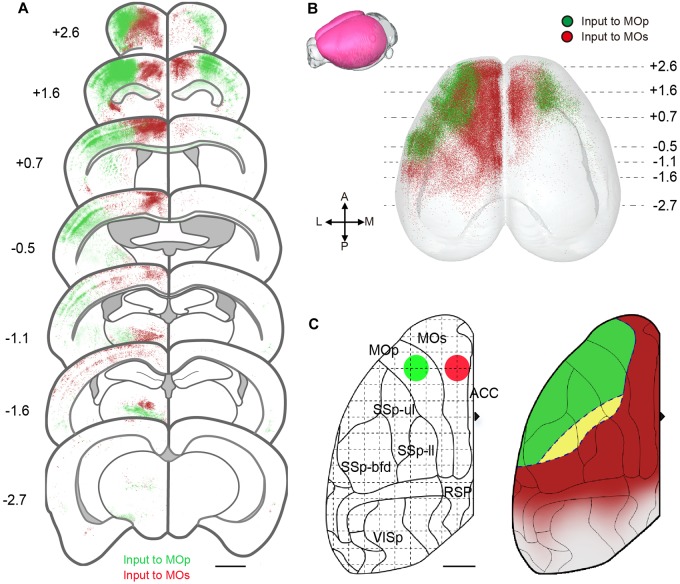
Region-specific cortical projection to MOp and MOs. **(A)** Coronal sections show inputs to the motor cortex from different cortical regions. The green signals indicate the neurons projecting to the Mop, while the red signals indicate the neurons projecting to the MOs. Scale bar = 1 mm. **(B)** Three-dimensional reconstruction of cortical areas. A dot denotes one neuron projecting to the MOp (green) or MOs (red). **(C)** Schematic diagram for input areas projecting to subregions of the MC. The green and red circles represent injection site positions. Green areas indicate regions mainly projecting to the Mop, while red areas indicate regions mainly projecting to the MOs; yellow areas indicate regions projecting to both the MOp and MOs. Scale bar = 1 mm.

To certify the difference between the input pattern of the MOp and MOs, we compared the distribution of input neurons in the individual brain, that was labeled with a dual-color RV. Using the whole brain dataset, we reconstructed the cortical areas in 3D. As shown in Figure 5, the cortex region is regionally specific for the MOp and MOs projection. The coronal slices exhibit a significant regional disjunction between the cortical inputs of subregions of the MC (Figure 5A). Based on the results of the 3D reconstruction (Figure 5B), we present a schematic diagram of the cortical projection to subregions of the MC (Figure 5C). In detail, the MOp mainly receives projections from the lateral portion of the ORB, the anterolateral portion of the MOs/MOp and the anterior portion of the SSp-bfd/SSs. The MOs mainly receives projections from the medial portion of the ORB/MOs, the posterior portion of the MOp/SSp-bfd/SSs/ACA and the anterior RSP/visual areas. The MOp and MOs both receive projections from the middle area of the SSp-bfd and SSp-ul/tr. The cortical circuits were divided into somatosensory motor subnetworks, medial subnetworks and lateral subnetworks in a previous study (Zingg et al., 2014). We found that the MOp mainly receives input from the lateral portion of the somatosensory motor subnetworks, while the MOs mainly receives input from the medial portion of the somatosensory motor subnetworks. The medial sub-networks have almost no projection to the MOp but has a large projection to the MOs, especially the ACA and the RSP in the second medial sub-network. The lateral subnetworks have a few projections to both the MOp and MOs.

**FIGURE 6:**
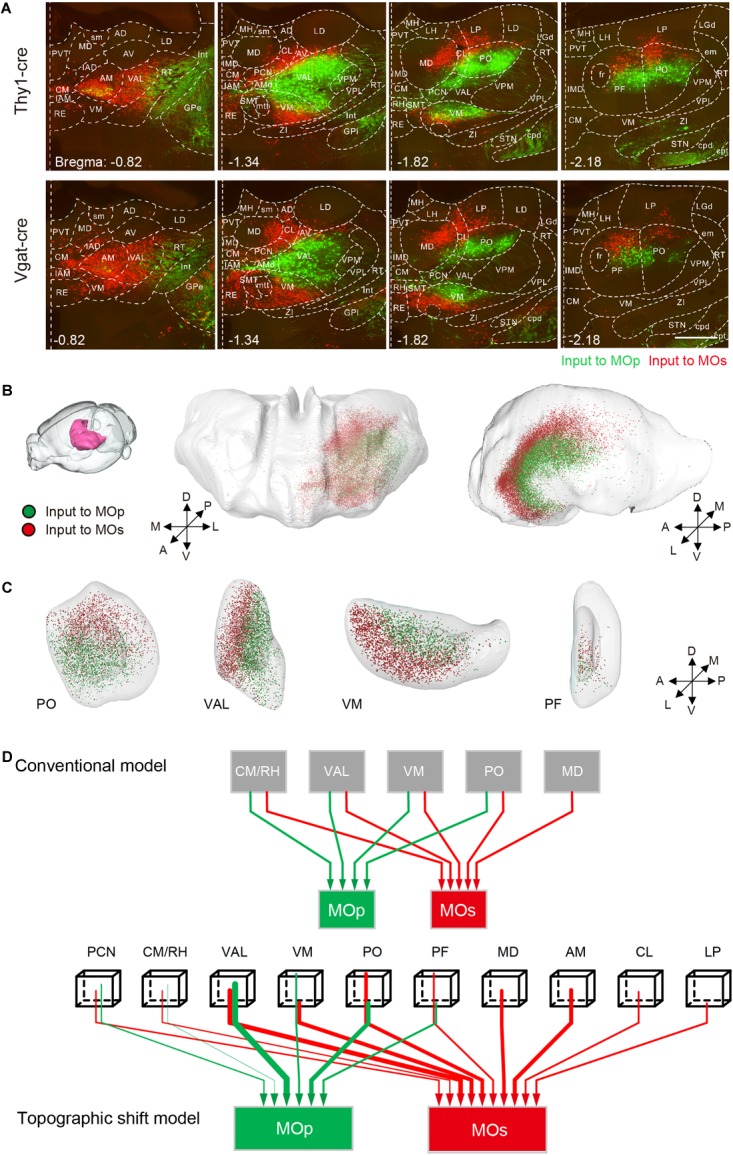
Region-specific thalamic projection to MOp and MOs. **(A)** Distribution of inputs to the MOp and MOs in the thalamus at different positions. The green signal indicates the neurons projecting to the MOp, while the red signal indicates the neurons projecting to the MOs. Scale bar = 500 μm. **(B)** Three-dimensional reconstruction of the thalamic areas. Green dots denote the thalamic neurons projecting to the MOp, while red dots denote the thalamic neurons projecting to the MOs. **(C)** Three-dimensional reconstruction of the PO, VAL, VM, and PF. **(D)** Schematic summary of the thalamic-cortical projection pattern. In the topographic shift model, the beginning tips of wirings indicate the relative spatial positions, and the thickness of wirings in both panels indicates the degree of projection.

The thalamus has a region-specific projection to the MOp and MOs as well. The results of the two-dimensional coronal plane exhibited that the AM, VAL, and the CM in the anterior part of the thalamus, have a certain number of projections to the MOs but not to the MOp. In the posterior portion of the thalamus, neurons projecting to the MOp gradually appear from the central part of the thalamus including the VAL, VM, PO, and the PF. While those neurons projecting to the MOs, gradually transfer to the dorsal and ventral sides including the VM, PO and the PF (Figure 6A and Supplementary Figure S7). With 3D reconstruction, the populations projecting to the MOp and MOs in the thalamus show an inner shell and an outer shell, respectively, while the outer shell encloses the inner shell (Figure 6B). In detail, the inner shell is mainly composed of the posterior portion of the VAL, the dorsal portion of the VM and the ventral portion of the PO/PF (Figure 6C). While the outer shell is mainly composed of the AM, the MD, the anterior portion of the VAL, the ventral portion of the VM and the dorsal portion of the PO/PF (Figure 6A,B). Based on the projection model of the thalamus to the MOp and MOs previously shown (Oh et al., 2014), we concluded a three-dimensional projection model with more information based on our data (Figure 6D).

**FIGURE 7:**
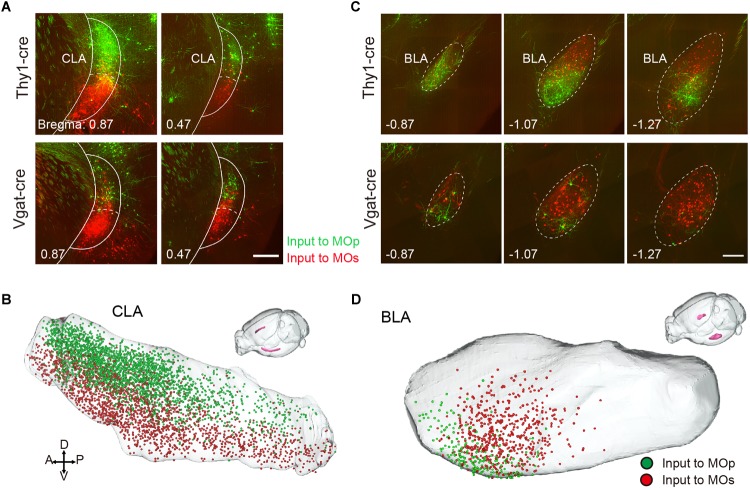
Region-specific projection from CLA and BLA. **(A)** Distribution of input neurons to the MOp and MOs in the CLA. The green signal indicates the neurons projecting to the Mop, while the red signal indicates the neurons projecting to the MOs. Scale bar = 500 μm. **(B)** Three-dimensional reconstruction of the CLA where green dots denote the neurons projecting to the MOp, while red dots denote the neurons projecting to the MOs. **(C)** Distribution of the input neurons to the MOp and MOs in the BLA. Scale bar = 500 μm. **(D)** Three-dimensional reconstruction of the BLA.

In other regions, the MOp and MOs also receive region-specific projections from the CLA and BLA. The neurons projecting to the MOp appear in the dorsal part of the CLA, while those projecting to the MOs focus in the ventral part of the CLA (Figure 7A,B). The neurons projecting to the MOp, and MOs focus in the ventral and dorsal part of the anterior BLA, respectively (Figure 7C,D). Although the projections from the basal forebrain regions to the MOp and MOs were not large, they showed regularity. In the MOp, almost no projections came from the anterior nucleus of the basal forebrain: the MS and the anterior part of the NDB. When we compared the distribution pattern into the basal forebrain (Figure 3), the ratio of projection to the MOp increased from the anterior parts to the posterior parts, as from the MS, NDB, SI to the PALd. These results were consistent with the results obtained in previous studies using retrograde fluorescent dyes (Zaborszky et al., 2015). Surprisingly, we found that the MOp receives a small number of projections from the contralateral cerebellar nuclei DN and the IP, with almost no projections to the MOs, and neither the MOp nor the MOs received projections from the ipsilateral DN and the IP. It has been thought that the cerebellum generally projects indirectly to the cortex, which mainly transmits information to the thalamus region as a relay station (Dum and Strick, 2003; Ramnani, 2006). Now, we are the first to reveal that the contralateral cerebellum nuclei can innervate the MOp directly.

## Discussion

Using a monosynaptic rabies tracing strategy, combined with continuously imaging using the fMOST system, we mapped the whole brain inputs to specific cell types in the subregions of the MC. We validated the afferent connections of the subregions of the MC revealed by previous studies, but with cell-type specificity and systematical comparison. The distribution patterns of inputs to the glutamatergic and GABAergic neurons in the MC are similar, which means that different cell types of individual brain regions receive inputs from the same areas. These results are in agreement with previous studies of the DR (Ogawa et al., 2014), the VTA (Beier et al., 2015), the SSp-bfd (Wall et al., 2016), the basal forebrain (Do et al., 2016), the dorsal striatum (Guo Q. et al., 2015). However, it is still unclear if these glutamatergic and GABAergic neurons are innervated by the same type of neurons and the same upstream circuits.

Importantly, using a 3D reconstruction, we found that the distribution of the input neurons that project to the primary and secondary MC are significantly different. In the cortex, the MOp is mainly innervated by the lateral portion of the somatosensory motor subnetworks, while the MOs is mainly innervated by medial portion of it. The medial subnetworks primarily project to the MOs instead of the MOp. Such a cortical connection pattern may indicate that, compared with the MOp, which mainly integrates somatosensory information to generate motion, the MOs integrates more sensory information (visual and auditory) and plays an important role in motor cognitive function (Sul et al., 2011; Murakami et al., 2014; Barthas and Kwan, 2017). In the thalamus, the projection from the VAL, VM, PO and the PF to subregions of the MC show regional specificity. The populations in the thalamus projecting to the MOp and MOs are like an inner shell and an outer shell respectively. Such a topographic relationship is similar to the distribution patterns of fibers in the thalamic areas, projecting from layer6 neurons in the MOp and MOs, respectively (Jeong et al., 2016). It verifies the reciprocal correspondence projection relationship between the subregions in the thalamus and cortex (Allendoerfer and Shatz, 1994; Hunnicutt et al., 2014; Oh et al., 2014). The motor thalamus nucleus, VAL and the VM, project to both the MOp and MOs, while the limbic thalamus, AM, and the MD, have a certain number of projections to the MOs but not to the MOp. These connections match the functional roles of the subregions of the MC, showing that the MOp may be involved in more motion generation and control, while the MOs is more inclined to be cognitively related to motion control. In addition, the connection patterns can provide a reference for the boundary demarcation of the thalamic nucleus (Hunnicutt et al., 2014) which is not clear in the cytoarchitecture.

Current results show that the neurons that project to the MOp, and MOs are regionally specific in the CLA and BLA. In previous studies on the projection from the CLA to the MOs and the ACA (Zingg et al., 2014), these results suggest that the CLA can be divided into different subregions that connect with different cortical areas with parallel and distinct circuits. The BLA has more projections to the MOs than to the MOp, which suggests that the BLA affects the excitability of the two subregions of the MC to varying degrees (Gokdemir et al., 2018). We found that the MOs receives large projections from the CA1 in the hippocampus. while the MOp does not. This may provide an anatomical reference that the MOs is involved in the regulation of spatial memory (Yamawaki et al., 2016). Here, we have not discussed the regions or populations innervating to the MOp and MOs simultaneously in the limitation of the dual-color RV labeling. In the technique, the rabies virus that was expressed in input neurons of the MOp in MOs, can get a supplement of the G protein in the MOs, then perform transsynaptic labeling of the MOs. In the single RV labeling experiments, the results showed that populations projected to the MOp, while the MOs segregated, which is consistent with the dual-color RV labeling.

In summary, we obtained a complete dataset of the inputs to specific cell types in the subregions of the MC. As far as we know, this study is the first to analyze and compare inputs to specific cell types in the subregions of the MC. Our results revealed a segregated but regional-specific projection pattern to the MOp and MOs. The distinct input patterns may be the root cause of functional differences between the MOp and MOs. Our results will be helpful in further understanding sophisticated brain connectivity and the function distinctions between the two subregions of the MC as well as lay a solid foundation to explore the behavioral impacts of them.

## Ethics Statement

All animal experiments were approved by the Animal Ethics Committee of Huazhong University of Science and Technology.

## Author Contributions

XL and HG conceived and designed the study. PL, YZ, and JT performed the experiments and analyzed the data. YH performed the whole-brain data acquisition. ZX and AL performed the whole-brain data processing. XL and PL wrote the manuscript.

## Conflict of Interest Statement

The authors declare that the research was conducted in the absence of any commercial or financial relationships that could be construed as a potential conflict of interest.

## References

[B1] Allendoerfer K. L. Shatz C. J. (1994). The subplate, a transient neocortical structure: its role in the development of connections between thalamus and cortex. *Annu. Rev. Neurosci.* 17 185–218. 10.1146/annurev.neuro.17.1.185 8210173

[B2] Barthas F. Kwan A. C. (2017). Secondary motor cortex: where ’Sensory’ Meets ’Motor’ in the rodent frontal cortex. *Trends Neurosci.* 40 181–193. 10.1016/j.tins.2016.11.006 28012708 PMC5339050

[B3] Beier K. T. Steinberg E. E. DeLoach K. E. Xie S. Miyamichi K. Schwarz L. (2015). Circuit architecture of VTA dopamine neurons revealed by systematic input-output mapping. *Cell* 162 622–634. 10.1016/j.cell.2015.07.015 26232228 PMC4522312

[B4] Campsall K. D. Mazerolle C. J. De Repentingy Y. Kothary R. Wallace V. A. (2002). Characterization of transgene expression and Cre recombinase activity in a panel of Thy-1 promoter-Cre transgenic mice. *Dev. Dyn.* 224 135–143. 10.1002/dvdy.10092 12112467

[B5] Chen C. Cheng M. Ito T. Song S. (2018). Neuronal organization in the inferior colliculus revisited with cell-type-dependent monosynaptic tracing. *J. Neurosci.* 38 3318–3332. 10.1523/JNEUROSCI.2173-17.2018 29483283 PMC6596054

[B6] Do J. P. Xu M. Lee S. H. Chang W. C. Zhang S. Chung S. (2016). Cell type-specific long-range connections of basal forebrain circuit. *eLife* 5:e13214. 10.7554/eLife.13214 27642784 PMC5095704

[B7] Dum R. P. Strick P. L. (2003). An unfolded map of the cerebellar dentate nucleus and its projections to the cerebral cortex. *J. Neurophysiol.* 89 634–639. 10.1152/jn.00626.2002 12522208

[B8] Ferreri F. Pasqualetti P. Maatta S. Ponzo D. Guerra A. Bressi F. (2011). Motor cortex excitability in Alzheimer’s disease: a transcranial magnetic stimulation follow-up study. *Neurosci. Lett.* 492 94–98. 10.1016/j.neulet.2011.01.064 21281700

[B9] Gokdemir S. Gunduz A. Ozkara C. Kiziltan M. E. (2018). Fear-conditioned alterations of motor cortex excitability: the role of amygdala. *Neurosci. Lett.* 662 346–350. 10.1016/j.neulet.2017.10.059 29097251

[B10] Gong H. Xu D. Yuan J. Li X. Guo C. Peng J. (2016). High-throughput dual-colour precision imaging for brain-wide connectome with cytoarchitectonic landmarks at the cellular level. *Nat. Commun.* 7:12142. 10.1038/ncomms12142 27374071 PMC4932192

[B11] Guo J. Z. Graves A. R. Guo W. W. Zheng J. Lee A. Rodriguez-Gonzalez J. (2015). Cortex commands the performance of skilled movement. *eLife* 4:e10774. 10.7554/eLife.10774 26633811 PMC4749564

[B12] Guo Q. Wang D. He X. Feng Q. Lin R. Xu F. (2015). Whole-brain mapping of inputs to projection neurons and cholinergic interneurons in the dorsal striatum. *PLoS One* 10:e0123381. 10.1371/journal.pone.0123381 25830919 PMC4382118

[B13] Hooks B. M. Mao T. Gutnisky D. A. Yamawaki N. Svoboda K. Shepherd G. M. (2013). Organization of cortical and thalamic input to pyramidal neurons in mouse motor cortex. *J. Neurosci.* 33 748–760. 10.1523/JNEUROSCI.4338-12.2013 23303952 PMC3710148

[B14] Hu R. Jin S. He X. Xu F. Hu J. (2016). Whole-brain monosynaptic afferent inputs to basal forebrain cholinergic system. *Front. Neuroanat.* 10:98. 10.3389/fnana.2016.00098 27777554 PMC5056182

[B15] Huang Z. J. (2014). Toward a genetic dissection of cortical circuits in the mouse. *Neuron* 83 1284–1302. 10.1016/j.neuron.2014.08.041 25233312 PMC4169123

[B16] Hunnicutt B. J. Long B. R. Kusefoglu D. Gertz K. J. Zhong H. Mao T. (2014). A comprehensive thalamocortical projection map at the mesoscopic level. *Nat. Neurosci.* 17 1276–1285. 10.1038/nn.3780 25086607 PMC4152774

[B17] Jeong M. Kim Y. Kim J. Ferrante D. D. Mitra P. P. Osten P. (2016). Comparative three-dimensional connectome map of motor cortical projections in the mouse brain. *Sci. Rep.* 6:20072. 10.1038/srep20072 26830143 PMC4735720

[B18] Kuan L. Li Y. Lau C. Feng D. Bernard A. Sunkin S. M. (2015). Neuroinformatics of the allen mouse brain connectivity atlas. *Methods* 73 4–17. 10.1016/j.ymeth.2014.12.013 25536338

[B19] Li X. Yu B. Sun Q. Zhang Y. Ren M. Zhang X. (2018). Generation of a whole-brain atlas for the cholinergic system and mesoscopic projectome analysis of basal forebrain cholinergic neurons. *Proc. Natl. Acad. Sci. U.S.A.* 115 415–420. 10.1073/pnas.1703601115 29259118 PMC5777024

[B20] Li Y. Gong H. Yang X. Yuan J. Jiang T. Li X. (2017). TDat: an efficient platform for processing petabyte-scale whole-brain volumetric images. *Front. Neural Circuits* 11:51. 10.3389/fncir.2017.00051 28824382 PMC5534480

[B21] Murakami M. Vicente M. I. Costa G. M. Mainen Z. F. (2014). Neural antecedents of self-initiated actions in secondary motor cortex. *Nat. Neurosci.* 17 1574–1582. 10.1038/nn.3826 25262496

[B22] Ogawa S. K. Cohen J. Y. Hwang D. Uchida N. Watabe-Uchida M. (2014). Organization of monosynaptic inputs to the serotonin and dopamine neuromodulatory systems. *Cell Rep.* 8 1105–1118. 10.1016/j.celrep.2014.06.042 25108805 PMC4142108

[B23] Oh S. W. Harris J. A. Ng L. Winslow B. Cain N. Mihalas S. (2014). A mesoscale connectome of the mouse brain. *Nature* 508 207–214. 10.1038/nature13186 24695228 PMC5102064

[B24] Osakada F. Mori T. Cetin A. H. Marshel J. H. Virgen B. Callaway E. M. (2011). New rabies virus variants for monitoring and manipulating activity and gene expression in defined neural circuits. *Neuron* 71 617–631. 10.1016/j.neuron.2011.07.005 21867879 PMC3189471

[B25] Peters A. J. Liu H. Komiyama T. (2017). Learning in the rodent motor cortex. *Annu. Rev. Neurosci.* 40 77–97. 10.1146/annurev-neuro-072116-031407 28375768 PMC5714655

[B26] Quan T. Zhou H. Li J. Li S. Li A. Li Y. (2016). NeuroGPS-Tree: automatic reconstruction of large-scale neuronal populations with dense neurites. *Nat. Methods* 13 51–54. 10.1038/nmeth.3662 26595210

[B27] Ramnani N. (2006). The primate cortico-cerebellar system: anatomy and function. *Nat. Rev. Neurosci.* 7 511–522. 10.1038/nrn1953 16791141

[B28] Ren M. Tian J. Zhao P. Luo J. Feng Z. Gong H. (2018). Simultaneous acquisition of multicolor information from neural circuits in resin-embedded samples. *Front. Neurosci.* 12:885. 10.3389/fnins.2018.00885 30555296 PMC6284031

[B29] Sanes J. N. Donoghue J. P. (2000). Plasticity and primary motor cortex. *Annu. Rev. Neurosci.* 23 393–415. 10.1146/annurev.neuro.23.1.393 10845069

[B30] Shepherd G. M. (2013). Corticostriatal connectivity and its role in disease. *Nat. Rev. Neurosci.* 14 278–291. 10.1038/nrn3469 23511908 PMC4096337

[B31] Sul J. H. Jo S. Lee D. Jung M. W. (2011). Role of rodent secondary motor cortex in value-based action selection. *Nat. Neurosci.* 14 1202–1208. 10.1038/nn.2881 21841777 PMC3164897

[B32] Tanji J. (2001). Sequential organization of multiple movements: involvement of cortical motor areas. *Annu. Rev. Neurosci.* 24 631–651. 10.1146/annurev.neuro.24.1.631 11520914

[B33] Tennant K. A. Adkins D. L. Donlan N. A. Asay A. L. Thomas N. Kleim J. A. (2011). The organization of the forelimb representation of the C57BL/6 mouse motor cortex as defined by intracortical microstimulation and cytoarchitecture. *Cereb. Cortex* 21 865–876. 10.1093/cercor/bhq159 20739477 PMC3059888

[B34] Vong L. Ye C. Yang Z. Choi B. Chua S Jr. Lowell B. B. (2011). Leptin action on GABAergic neurons prevents obesity and reduces inhibitory tone to POMC neurons. *Neuron* 71 142–154. 10.1016/j.neuron.2011.05.028 21745644 PMC3134797

[B35] Wall N. R. De La Parra M. Sorokin J. M. Taniguchi H. Huang Z. J. Callaway E. M. (2016). Brain-wide maps of synaptic input to cortical interneurons. *J. Neurosci.* 36 4000–4009. 10.1523/JNEUROSCI.3967-15.2016 27053207 PMC4821911

[B36] Wall N. R. Wickersham I. R. Cetin A. De La Parra M. Callaway E. M. (2010). Monosynaptic circuit tracing in vivo through Cre-dependent targeting and complementation of modified rabies virus. *Proc. Natl. Acad. Sci.* 107 21848–21853. 10.1073/pnas.1011756107 21115815 PMC3003023

[B37] Wickersham I. R. Finke S. Conzelmann K. K. Callaway E. M. (2007a). Retrograde neuronal tracing with a deletion-mutant rabies virus. *Nat. Methods* 4 47–49. 10.1038/nmeth999 17179932 PMC2755236

[B38] Wickersham I. R. Lyon D. C. Barnard R. J. O. Mori T. Finke S. Conzelmann K.-K. (2007b). Monosynaptic restriction of transsynaptic tracing from single, genetically targeted neurons. *Neuron* 53 639–647. 10.1016/j.neuron.2007.01.033 17329205 PMC2629495

[B39] Yamawaki N. Radulovic J. Shepherd G. M. (2016). A corticocortical circuit directly links retrosplenial cortex to M2 in the mouse. *J. Neurosci.* 36 9365–9374. 10.1523/JNEUROSCI.1099-16.2016 27605612 PMC5013186

[B40] Yang H. Yang J. Xi W. Hao S. Luo B. He X. (2016). Laterodorsal tegmentum interneuron subtypes oppositely regulate olfactory cue-induced innate fear. *Nat. Neurosci.* 19 283–289. 10.1038/nn.4208 26727549

[B41] Yang Y. Wang Z.-H. Jin S. Gao D. Liu N. Chen S.-P. (2016). Opposite monosynaptic scaling of BLP–vCA1 inputs governs hopefulness- and helplessness-modulated spatial learning and memory. *Nat. Commun.* 7:11935. 10.1038/ncomms11935 27411738 PMC4947155

[B42] Zaborszky L. Csordas A. Mosca K. Kim J. Gielow M. R. Vadasz C. (2015). Neurons in the basal forebrain project to the cortex in a complex topographic organization that reflects corticocortical connectivity patterns: an experimental study based on retrograde tracing and 3D reconstruction. *Cereb. Cortex* 25 118–137. 10.1093/cercor/bht210 23964066 PMC4259277

[B43] Zhang S. Xu M. Chang W. C. Ma C. Hoang Do J. P. Jeong D. (2016). Organization of long-range inputs and outputs of frontal cortex for top-down control. *Nat. Neurosci.* 19 1733–1742. 10.1038/nn.4417 27749828 PMC5127741

[B44] Zingg B. Hintiryan H. Gou L. Song M. Y. Bay M. Bienkowski M. S. (2014). Neural networks of the mouse neocortex. *Cell* 156 1096–1111. 10.1016/j.cell.2014.02.023 24581503 PMC4169118

