# Supplementary material for: Whole Brain Mapping of Long-Range Direct Input to Glutamatergic and GABAergic Neurons in Motor Cortex
Source: Front Neuroanat. 2019 Apr 17;13:44. doi: 10.3389/fnana.2019.00044 (PMC6478816; doi:10.3389/fnana.2019.00044)
Supplement: Supplementary file 1 [file Table_1.DOCX]

**Supplementary Table 1 Abbreviation for brain area**

| **Abbreviation for brain area** | |
| --- | --- |
| Agranular insular area/Gustatory | AI/GU |
| Anterior cingulate area | ACA |
| Anteromedial nucleus of thalamus | AM |
| Anteromedial visual area | VISl |
| Auditory areas | AUD |
| Basolateral amygdala nucleus | BLA |
| Central lateral nucleus of thalamus | CL |
| Central medial nucleus of thalamus | CM |
| Cerebellar nuclei | CBN |
| Claustrum | CLA |
| Contralateral dentate nucleus/Interposed nucleus | con-DN/IP |
| Contralateral primary motor area | con-MOp |
| Contralateral secondary motor area | con-MOs |
| Cortical plate | CTXpl |
| Cortical subplate | CTXsp |
| Diagonal band nucleus | NDB |
| Dorsal nucleus raphe | DR |
| Dorsal peduncular area/Taenia tecta | DP/TT |
| Entorhinal area | ENT |
| Gigantocellular/Intermediate reticular nucleus | GRN/IRN |
| Hippocampal region | HIP |
| Infralimbic area | IL |
| Lateral posterior nucleus of thalamus | LP |
| Lateral visual area | VISl |
| Medial septal nucleus | MS |
| Mediodorsal nucleus of thalamus | MD |
| Medulla | MY |
| Midbrain, behavioral state related | MBsta |
| Midbrain, motor related | MBmot |
| Midbrain reticular nucleus | MRN |
| Orbital area, lateral part | ORBl |
| Orbital area, ventrolateral part | ORBvl |
| Orbital area, medial part | ORBm |
| Pallidum | PAL |
| Pallidum, dorsal region | PALd |
| Paracentral nucleus of thalamus | PCN |
| Parafascicular nucleus of thalamus | PF |
| Pedunculopontine nucleus | PPN |
| Pons | P |
| Pontine gray | PG |
| Posterior complex of thalamus | PO |
| Prelimbic area | PL |
| Primary motor cortex | MOp |
| Primary somatosensory area, barrel field | SSp-bfd |
| Primary somatosensory area, lower limb/trunk | SSp-ll/tr |
| Primary somatosensory area, upper limb/mouth | SSp-ul/m |
| Primary visual area | VISp |
| Retrosplenial area | RSP |
| Secondary motor cortex | MOs |
| Substantia innominata | SI |
| Substantia nigra, reticular part | SNr |
| Superior central nucleus raphe | CS |
| Superior colliculus | SC |
| Supplemental somatosensory area | SSs |
| Temporal association/Ectorhinal/Perirhinal areas | TEa/ECT/PERI |
| Thalamus | TH |
| Ventral anterior-lateral complex of thalamus | VAL |
| Ventral medial nucleus of thalamus | VM |
| Ventral tegmental area | VTA |


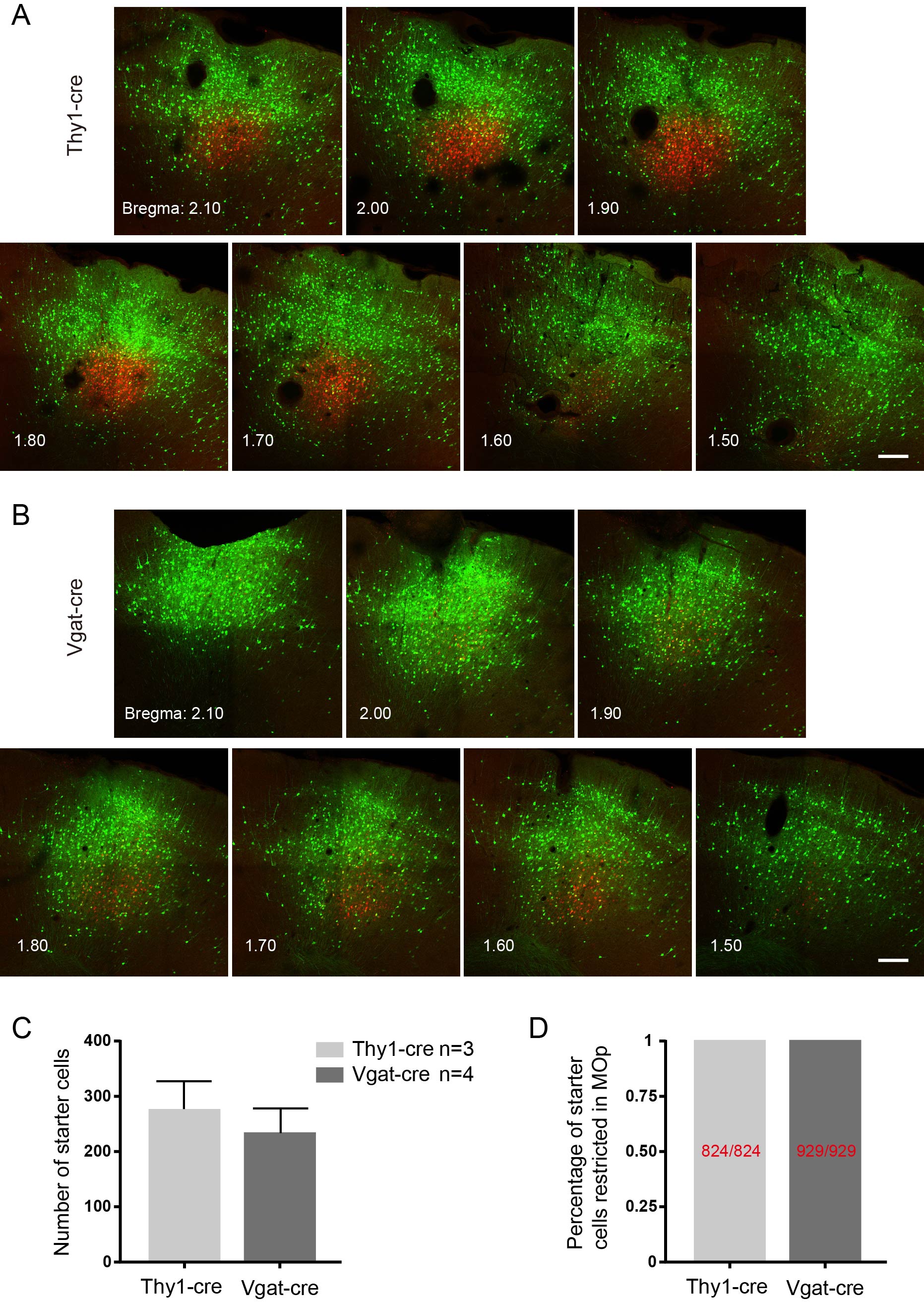


**Figure S1. Continuous presentation around the injection sites.**

Here, we show continuous coronal figure around the injection sites in thy1-cre and Vgat-cre samples which are labeled by rAAV2/9-Ef1α-DIO-mcherry-2a-TVA-WPRE-pA, rAAV2/9- Ef1α-DIO-RG-WPRE-pA and RV-△G-EnVA-EGFP in MOp. (A) Thy1-cre mice, scale bar=200μm. (B) Vgat-cre mice, scale bar=200μm. (C) Number of starter cells (Thy1-cre, n=3; Vgat-cre, n=4). (D) Percentage of starter cells restricted in MOp.

**
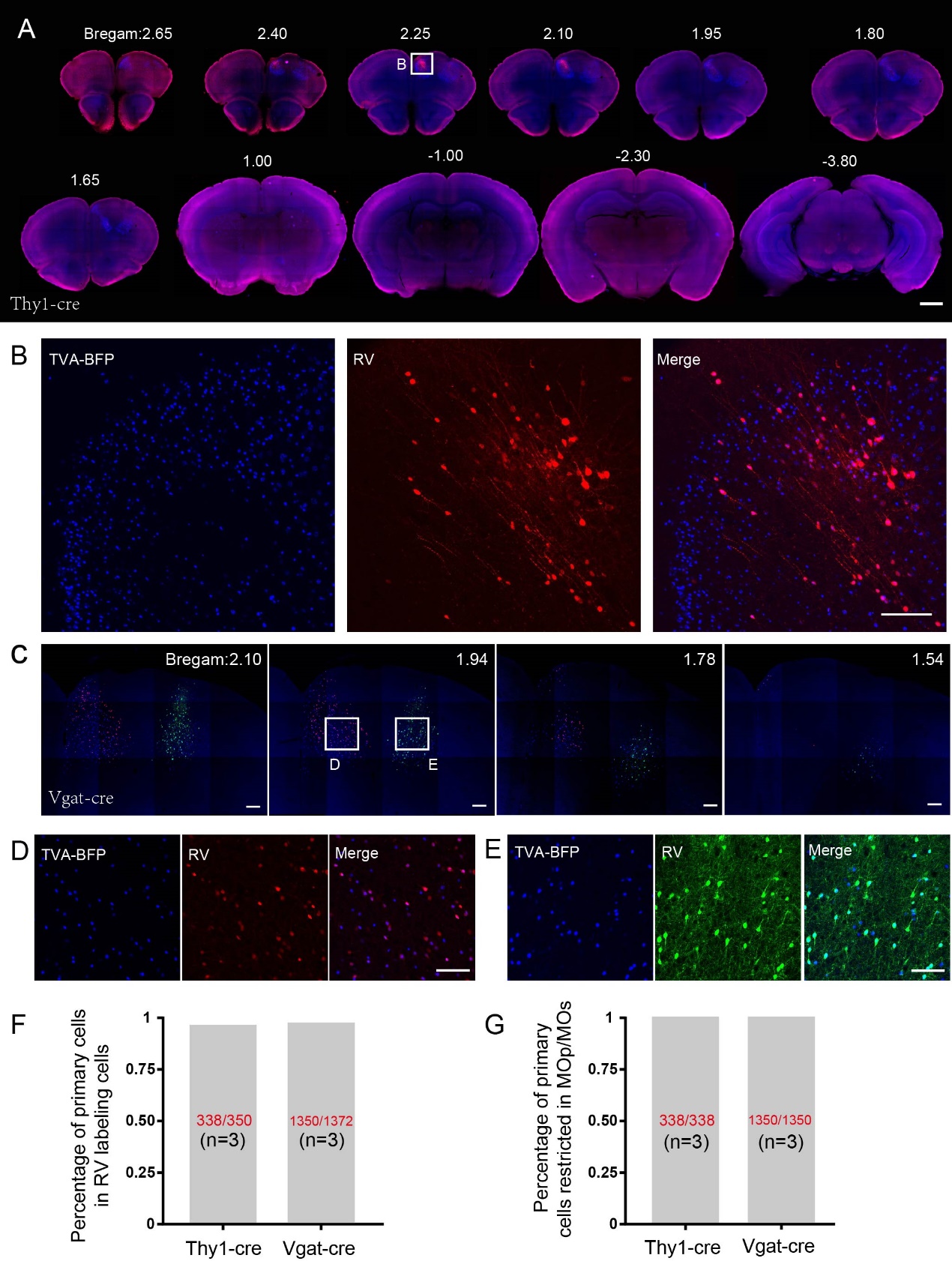
**

**Figure S2. Control experiments for monosynaptic rabies tracing strategy without RG.**

Here, we injected TVA-BFP and RV without RG in MOp and MOs to check the distribution of primary cells (before trans-synaptic infection). In Thy1-cre mice, we injected RV-△G-EnVA-DsRed in MOs. In Vgat-cre mice, we injected RV-△G-EnVA-DsRed in MOs and RV-△G-EnVA-EGFP in MOp. (A) Coronals near the injection site and at other site in Thy1-cre mice. Scale bar=1000μm. (B) Details of the white box in (A). Scale bar=100μm. (C) Coronals near the injection site in Vgat-cre mice. Scale bar=200μm. (D) and (E) Details of the white box in (C). Scale bar=100μm. (F) Percentage of primary cells in RV labeling cells (Thy1-cre: in total, n=3; Vgat-cre: in total, n=3). (G) Percentage of primary cells restricted in MOp and MOs.


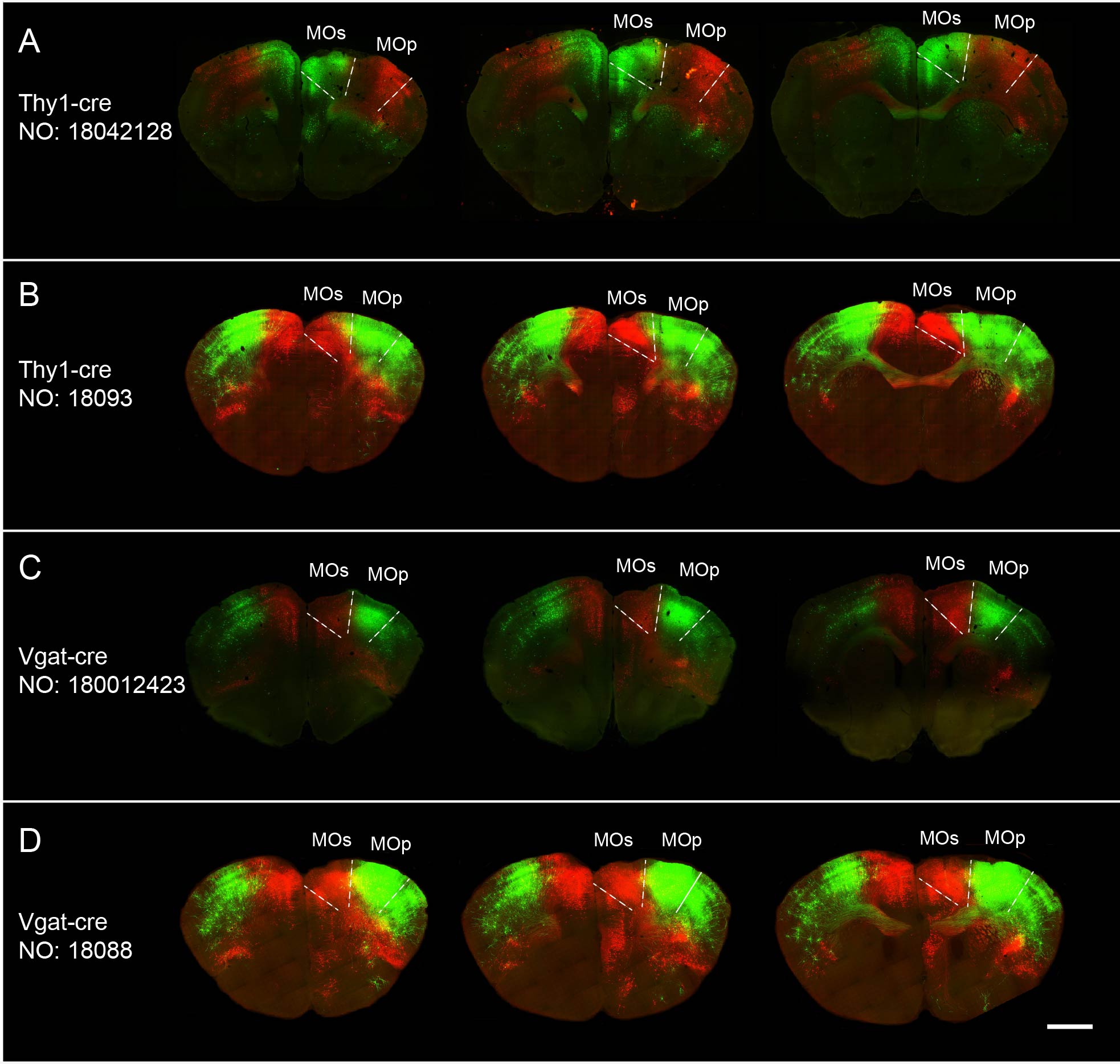


**Figure S3. The infected cells restricted into the injected brain region.**

Here, we show continuous coronals near injection sites of two thy1-cre and two Vgat-cre samples. (A) and (B) Thy1-cre mice. (C) and (D) Vgat-cre mice. Scale bar=1mm.


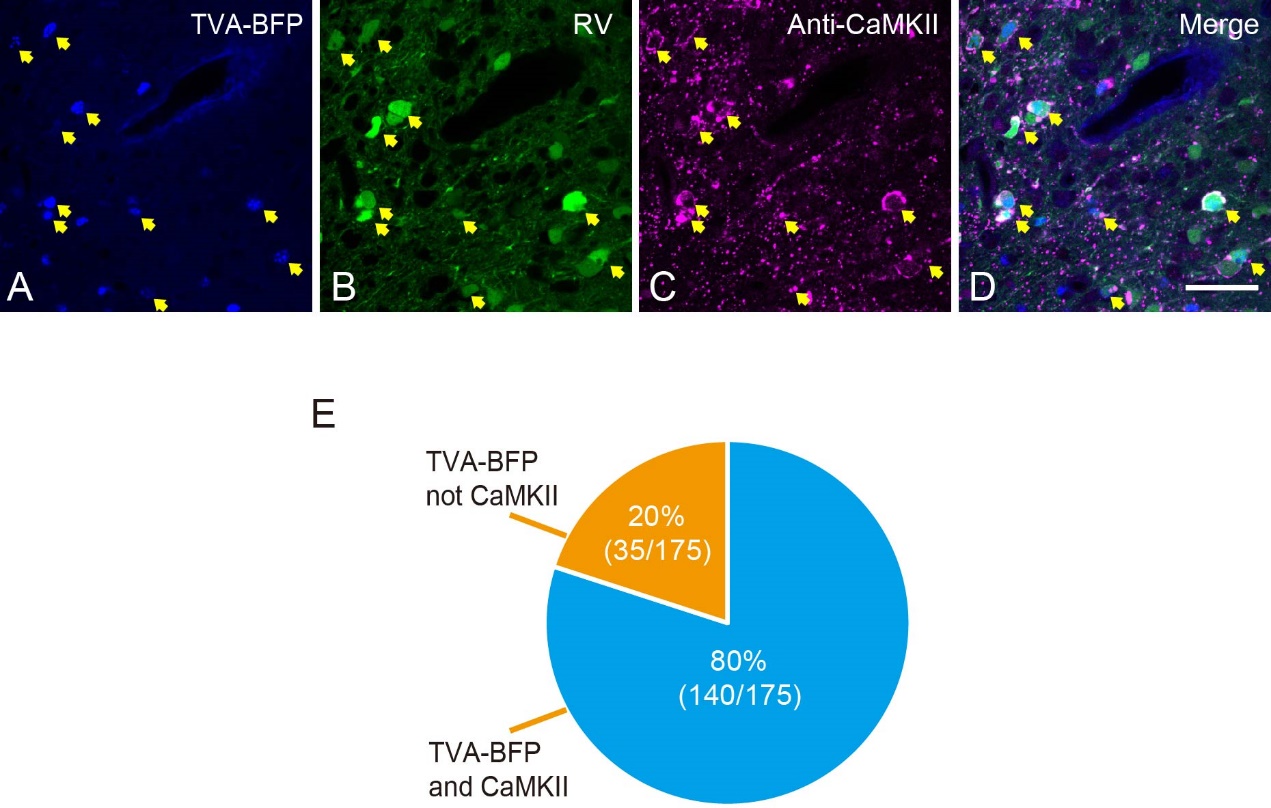


**Figure S4. Immunohistochemical staining for glutamatergic neurons.**

We performed immunohistochemical staining for the marker CaMKII (Abcam ab5683) at the MOp injection site of thy1-cre samples. (A-D) Immunohistochemical staining for glutamatergic neurons. Scale bar=50μm. (E) Percentage of TVA-BFP+CaMKII normalized with TVA-BFP.


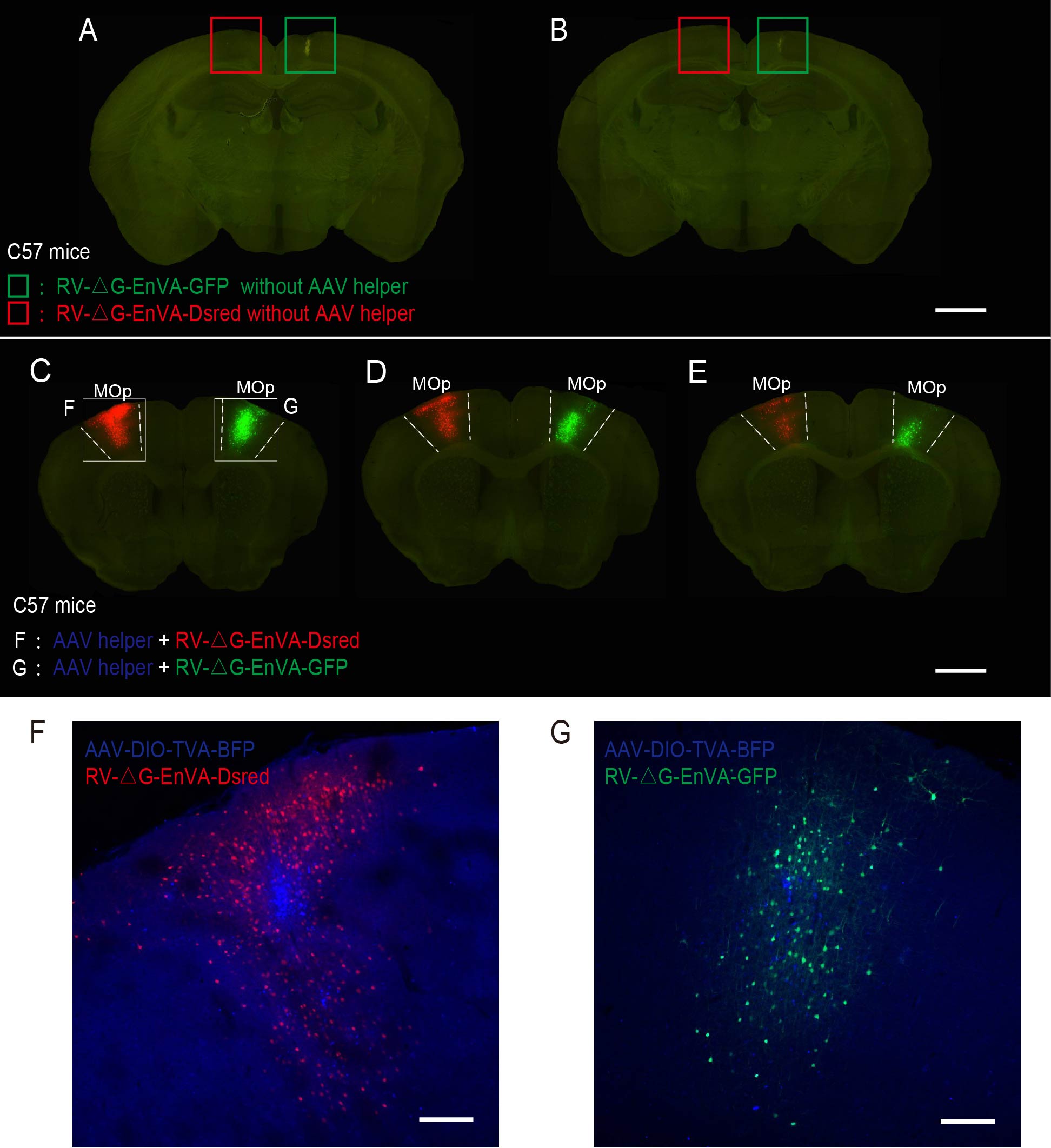


**Figure S5. Control experiments for virus expression in C57BL/6J mice.**

In control experiments, the RVs expressed different fluorescent proteins (Left: RV-△G-EnVA-DsRed; Right: RV-△G-EnVA-EGFP) were injected into the bilateral parietal cortex of C57BL/6J mice. In another group of experiments, the Cre-dependent AAV helper expressed TVA-BFP and RG were injected into the bilateral MOp (AP: 1.54mm, ML: ±1.70mm, DV: -1.50mm) in C57BL/6J mice. Three weeks later, RVs expressed different fluorescent proteins (Left: RV-△G-EnVA-DsRed; Right: RV-△G-EnVA-EGFP) were injected into these sites respectively.

(A-B) Continuous coronal slices of the injection site of C57BL/6J mice control for RV-△G-EnVA-DsRed and RV-△G-EnVA-EGFP, and the red box and green box indicate the injection site of RVs expressed Dsred and GFP respectively. (C-E) Continuous coronal slices of the injection site of C57BL/6J mice control for monosynaptic rabies tracing system. (F-G) Detail signals of TVA-BFP and RV-XFP in (C). Scale bar: A-E, 1 mm; F-G, 200μm.


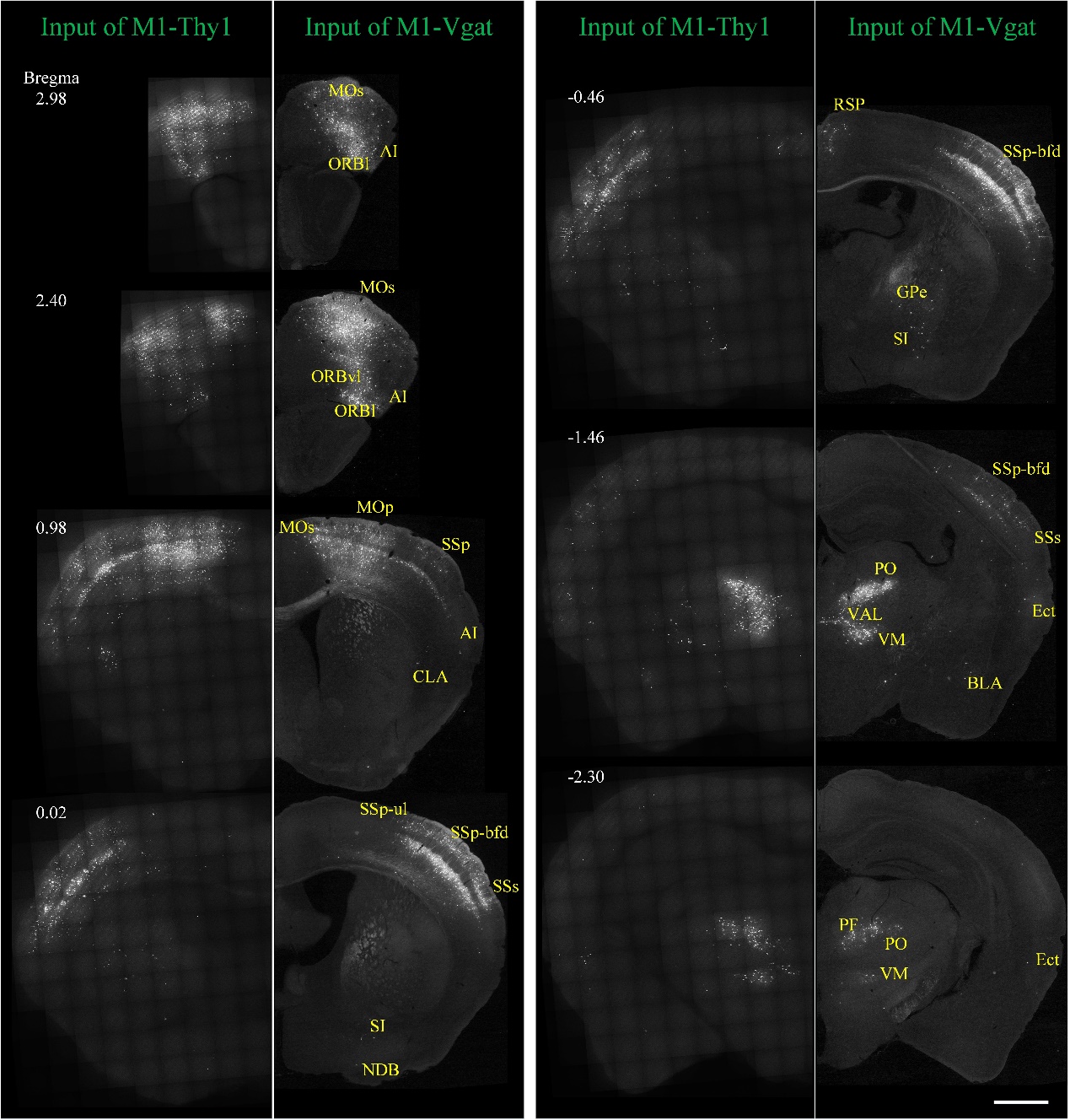


**Figure S6.** **Slices of ipsilateral hemispheres of the injection site labeled by single RV.**

Here, we show coronals across the whole brain of Thy1-cre and Vgat-cre samples which were labeled by single RV in MOp. Scale bar=1mm.


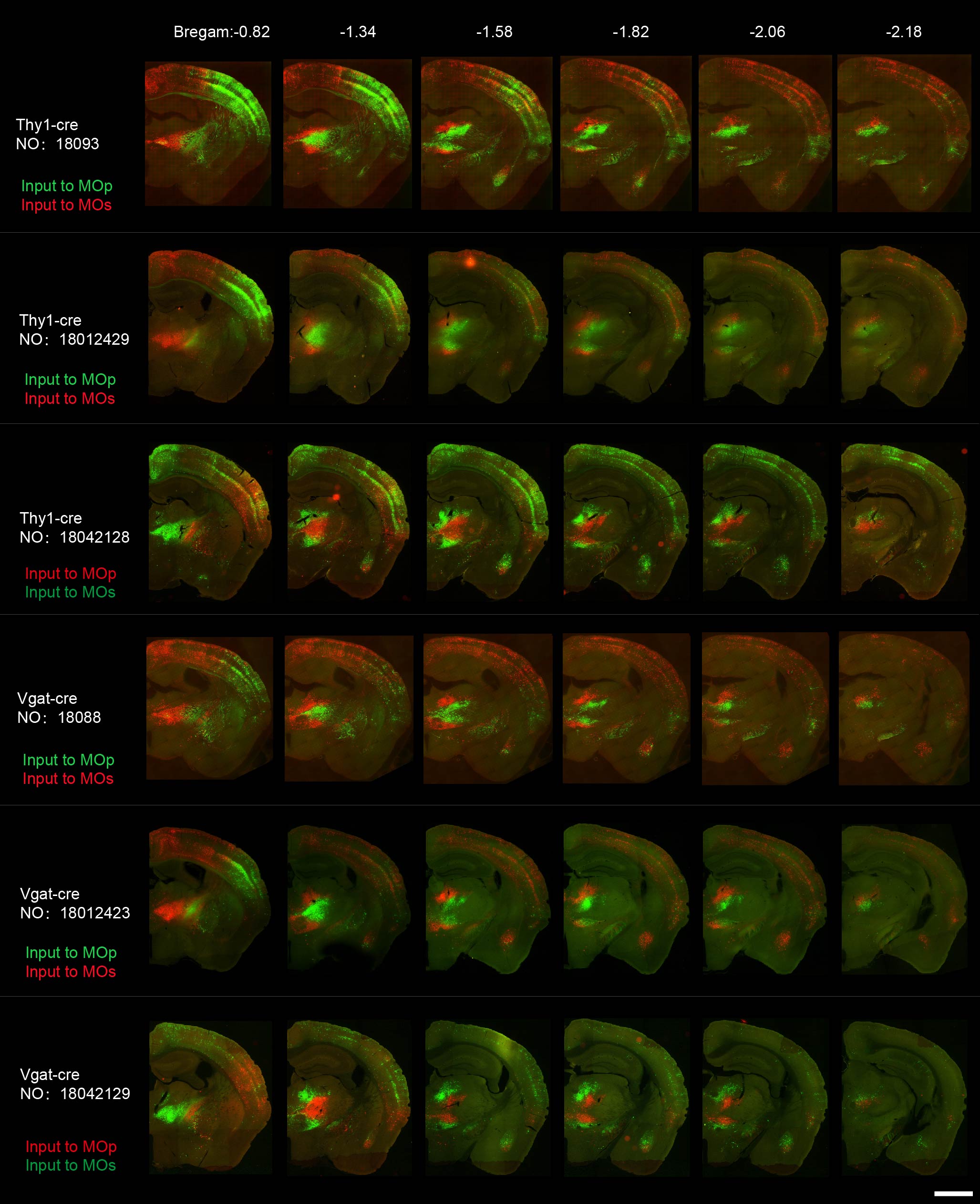


**Figure S7.** **Display of thalamic signal distribution of 6 samples.**

Here, we show the continuous coronals to show the separated distribution of thalamic populations projecting to MOp and MOs respectively. Scale bar=1mm.
